# FReSCO: Flow Reconstruction and Segmentation for low‐latency Cardiac Output monitoring using deep artifact suppression and segmentation

**DOI:** 10.1002/mrm.29374

**Published:** 2022-07-04

**Authors:** Olivier Jaubert, Javier Montalt‐Tordera, James Brown, Daniel Knight, Simon Arridge, Jennifer Steeden, Vivek Muthurangu

**Affiliations:** ^1^ UCL Center for Translational Cardiovascular Imaging University College London London UK; ^2^ Department of Computer Science University College London London UK; ^3^ Department of Cardiology Royal Free London NHS Foundation Trust London UK

**Keywords:** Cardiac MRI, deep learning, Cardiac output, monitoring, real‐time, Flow imaging

## Abstract

**Purpose:**

Real‐time monitoring of cardiac output (CO) requires low‐latency reconstruction and segmentation of real‐time phase‐contrast MR, which has previously been difficult to perform. Here we propose a deep learning framework for “FReSCO” (Flow Reconstruction and Segmentation for low latency Cardiac Output monitoring).

**Methods:**

Deep artifact suppression and segmentation U‐Nets were independently trained. Breath‐hold spiral phase‐contrast MR data (*N* = 516) were synthetically undersampled using a variable‐density spiral sampling pattern and gridded to create aliased data for training of the artifact suppression U‐net. A subset of the data (*N* = 96) was segmented and used to train the segmentation U‐net. Real‐time spiral phase‐contrast MR was prospectively acquired and then reconstructed and segmented using the trained models (FReSCO) at low latency at the scanner in 10 healthy subjects during rest, exercise, and recovery periods. Cardiac output obtained via FReSCO was compared with a reference rest CO and rest and exercise compressed‐sensing CO.

**Results:**

The FReSCO framework was demonstrated prospectively at the scanner. Beat‐to‐beat heartrate, stroke volume, and CO could be visualized with a mean latency of 622 ms. No significant differences were noted when compared with reference at rest (bias = −0.21 ± 0.50 L/min, *p* = 0.246) or compressed sensing at peak exercise (bias = 0.12 ± 0.48 L/min, *p* = 0.458).

**Conclusions:**

The FReSCO framework was successfully demonstrated for real‐time monitoring of CO during exercise and could provide a convenient tool for assessment of the hemodynamic response to a range of stressors.

## Abstract 2

Click here for author‐reader discussions

## 1 INTRODUCTION

Continuous assessment of cardiac output (CO) has several applications such as evaluating the response to exercise or pharmacological innervations.^1^, ^2^, ^3^, ^4^ The noninvasive reference standard method of measuring CO is phase‐contrast MR (PCMR).^5^, ^6^, ^7^ However, conventional PCMR uses segmented k‐space acquisitions, and therefore cannot be used to continuously monitor CO. Real‐time PCMR can be used for this application, but ensuring adequate spatiotemporal resolution requires significant data undersampling, often combined with efficient trajectories (eg, spiral).^8^

Unfortunately, reconstruction of highly accelerated non‐Cartesian data is time‐consuming due to the iterative nature of state‐of‐the art reconstruction algorithms (eg, compressed sensing [CS]). This problem has been partly mitigated by graphics processing units that enable low‐latency reconstruction of continuously acquired real‐time data.^9^ However, processing the large amounts of data produced is time‐consuming, and current methods do not enable real‐time monitoring.

We have recently shown that deep learning (DL) can be used to remove aliasing artifact (deep artifact suppression) from both magnitude and phase images of highly accelerated real‐time spiral PCMR data acquired at rest.^10^ The U‐Net architecture used for deep artifact suppression was originally proposed for segmentation of biomedical images^11^ and performed excellently for a wide range of segmentation applications.^12^ We propose extending our previous work^10^ by performing both low‐latency deep artifact suppression and segmentation on real‐time flow data and combining this with optimized communication and visualization for near real‐time monitoring of CO during exercise. The specific aims were to (1) develop and demonstrate the feasibility of a low‐latency continuous CO monitoring framework on the scanner in 10 healthy subjects during a simple exercise study, (2) compare CO measurements at rest between our proposed “FReSCO” (Flow Reconstruction and Segmentation for low latency CO monitoring) method and reference free‐breathing retrospectively electrocardiogram (ECG)–gated Cartesian PCMR, and (3) compare CO measurements at rest and peak exercise between FReSCO and CS reconstruction.

## 2 METHODS

**FIGURE 1:**
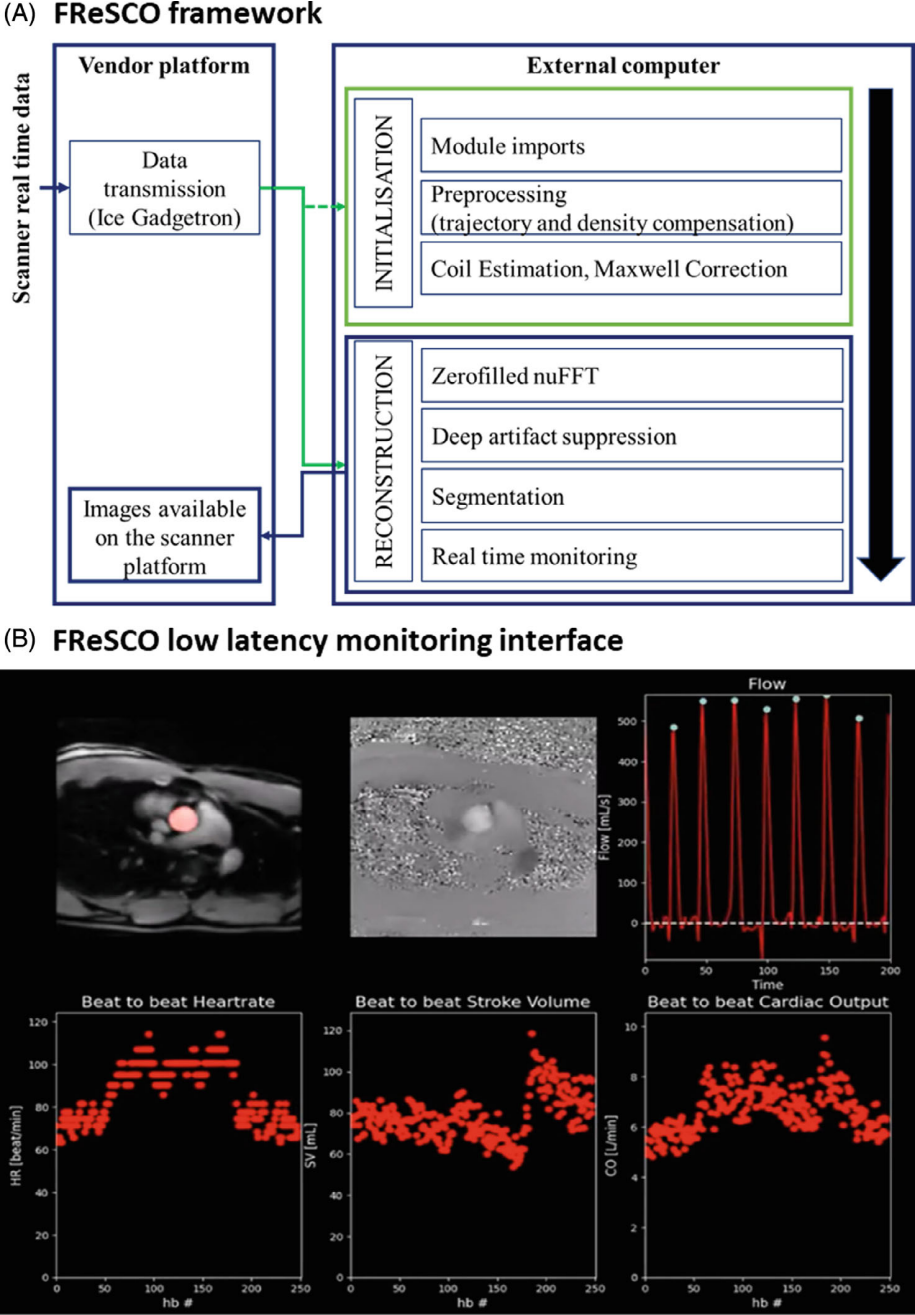
The “FReSCO” (Flow Reconstruction and Segmentation for low latency Cardiac Output monitoring) framework for low‐latency monitoring of aortic flow. A, Real‐time golden‐angle variable density spiral data are forwarded to an external computer using Gadgetron. The pipeline is initialized using the 10 first frames (eg, module imports, trajectory, density compensation, coil estimation, Maxwell correction), and the proposed reconstruction and flow monitoring are performed during scanning. Flow maps and segmentations are sent back to the scanner. B, Illustration of the proposed real‐time monitoring interface at the end of a 3‐min exercise scan. Top left: segmented magnitude images; top middle: phase images; top right: flow curves; bottom left: beat‐to‐beat heartrate; bottom middle: stroke volume; bottom right: cardiac output. Abbreviation: nuFFT, nonuniform fast Fourier transform

Our proposed framework for low‐latency CO monitoring relies on (1) a highly accelerated real‐time spiral PCMR acquisition, (2) an open‐source cross‐platform communication framework (Gadgetron), and (3) two sequential U‐Nets for fast deep artifact suppression and segmentation of the real‐time data. The FReSCO framework is illustrated in Figure 1. Cohort and acquisition information relative to the prospective and retrospective studies is summarized in Supporting Information Table S1.

### 2.1 Acquisition

The real‐time flow acquisition uses a golden‐angle variable density spiral^13^ trajectory, with the outer 10% of k‐space 2.5× less densely sampled than the inner 20% (with linearly decreasing density in between [trajectory depicted in Supporting Information Figure S1]). One‐sided velocity encoding was achieved by acquiring each readout twice (velocity encoded and compensated), and three spiral interleave positions (six readouts) were acquired per frame, leading to an acceleration factor of about 8.7/21.7 for inner/outer k‐space. Scan parameters included FOV = 400 × 400 mm, voxel size = 2.1 × 2.1 × 6.0 mm, TR/TE = 5.8/2.1 ms, temporal resolution = 35.0 ms, flip angle = 20°, and velocity encoding = 200 cm/s.

Reference standard flow imaging was acquired per our clinical protocol with high spatial/temporal resolution (enabled through cardiac gating and respiratory averaging) to assess accuracy of our method at rest. It consists of a free‐breathing, retrospectively ECG‐gated, Cartesian PCMR sequence with the following parameters: FOV = 254 × 370 mm, voxel size = 1.4 × 1.4 × 6.0 mm, TR/TE = 5.1/2.7 ms, temporal resolution ∼ 30 ms, flip angle = 20°, velocity encoding = 180 cm/s, averages = 3, and acquisition time = 95.71 ± 21.4 s.

### 2.2 Training data

The FReSCO DL framework consisted of independently trained deep artifact suppression and aortic segmentation networks. The training data for deep artifact suppression were created from 516 breath‐hold, retrospectively ECG‐gated, uniform density spiral PCMR^8^ data sets (Supporting Information Table S1) in the aortic position of patients with pediatric and/or congenital heart disease (age: 32.9 ± 14.0 years, heartrate: 74.3 ± 15.3 bpm). Each data set consisted of magnitude and phase‐subtracted images (as stored within clinical routine). To create the paired synthetically corrupted and truth images for training, the complex data were first Fourier‐transformed and undersampled with the proposed trajectory. The synthetic undersampled k‐space data were then inverse Fourier–transformed to produce the aliased images. This deep artifact suppression data set was split into 470/30/16 for training, validation, and testing.

The training data for segmentation were created from 96 of these data sets (age: 20.9 ± 13.5 years, heartrate: 74.5 ± 15.9 bpm) using a semi‐automatic method based on an optical flow registration with manual operator correction^14^ by an expert (V.M.). This segmentation data set (complex images as input and segmentation masks as output) was split into 70/10/16 for training, validation, and testing (with the same test set as used for the artifact suppression network).

Collection of all retrospective data was approved by the national research ethics committee (Ref. 06/Q0508/124).

### 2.3 Networks and training

**FIGURE 2:**
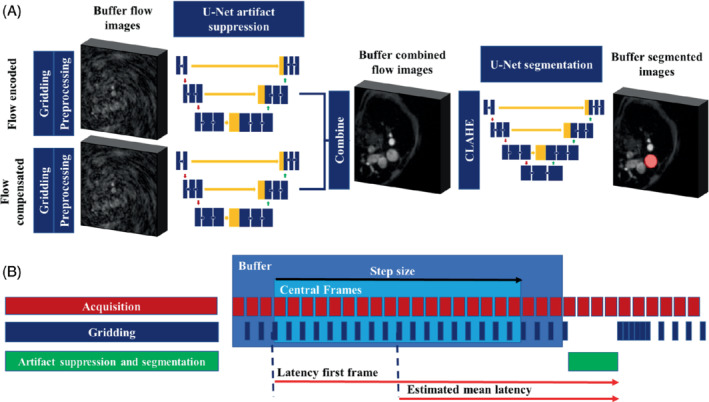
A, Overview of the FReSCO framework at inference. Deep artifact suppression is performed on the buffered gridded flow‐encoded and compensated data. Segmentation is performed on the combined magnitude contrast limited adaptive histogram equalized (CLAHE) images. B, Sliding window reconstruction timings (at scale). Each acquired frame is gridded independently but deep artifact–suppressed and segmented as a block of 24 frames

The overall DL framework for deep artifact suppression and segmentation is shown in Figure 2A and consisted of two consecutive, independently trained, 3D U‐Nets. The artifact suppression model was trained on center‐cropped (128 × 128), paired complex corrupted and truth images in blocks of 24 frames, with real and imaginary channels, using an average 2D structural similarity index (SSIM) based loss (L)^10^:

$$L=1-\frac{\text{SSIM}\left(\;\frac{\text{real}\left(y\right)+1}{2},\frac{\text{real}\left(\hat{y}\right)+1}{2}\;\right)+\text{SSIM}\left(\frac{\text{imag}\left(y\right)+1}{2},\frac{\text{imag}\left(\hat{y}\right)+1}{2}\right)\;}{2}$$

where y is the ground‐truth image; $\hat{y}$ is the predicted image; and real and imag are the real and imaginary components. Data augmentation was performed during training, and included random smooth phase offsets, image flips, rotations, and roll (ie, time shift). In addition, translational motion was applied to 50% of cases to simulate exercise and rest cases. A Hyperband optimization^15^ was performed to choose the optimum U‐Net parameters, including number of scales, initial filters, convolution blocks per scale, and learning rate (the tunable U‐Net architecture is depicted in Supporting Information Figure S2 and range of parameters explored in Supporting Information Table S2).

The segmentation model was also trained on 24 frames of cropped (128 × 128) paired complex images (as real and imaginary channels) and segmentation maps. The magnitude data underwent contrast limited adaptive histogram equalization (CLAHE)^16^ for more robust segmentation. Data augmentation and hyperparameter optimization were broadly the same as in the artifact suppression model, except that the type of segmentation loss was also optimized (as described in Supporting Information Table S2).

Hyperband parameters for the deep artifact suppression/segmentation models included 100/150 maximum number of epochs, hyperband factor of 3 (discarded proportion within each bracket), and 172 of 172 configurations tested. All training was performed using TensorFlow^17^ on a Linux Workstation (with NVIDIA TITAN RTX 24GB).

### 2.4 In silico validation

Evaluation of both tasks was performed on the test set (including motion in all 16 cases) using (1) imaging metrics (mean absolute error, peak SNR, and average SSIM) and (2) segmentation metrics (binary cross entropy and Dice score). The segmentation network's performance was tested on both the original “truth” test images and on the DL restored corrupted images to evaluate any loss in performance due to the DL reconstruction.

### 2.5 Prospective experiments

Ten healthy subjects (age: 33.2 ± 4.3 years) were prospectively acquired (Aera 1.5 T; Siemens Healthineers). Reference standard resting aortic PCMR data were acquired using a retrospectively ECG‐gated, breath‐hold cartesian PCMR scan followed by 3 min of continuous real‐time imaging (5143 frames) during rest, exercise, and recovery.

A simple exercise protocol was used to demonstrate feasibility of the framework consisting of 40 s of rest, 80 s of moderate exercise, and 60 s of recovery. The exercise consisted of supine repeated leg extensions (following a metronome at 1 beat per second) using an extensible band held by the subject to provide resistance.

Collection of prospective data was approved by the national research ethics committee (Ref. 17/LO/1499), and written consent was obtained in all subjects.

### 2.6 In vivo real‐time reconstruction

Prospective images were reconstructed in near‐real time during scanning using Gadgetron^18^ for low‐latency communication with an external reconstruction and visualization computer (Linux Workstation with NVIDIA GeForce RTX 3060 12GB). TensorFlow MRI^19^ was used for gridding, and the proposed networks were used for deep artifact suppression and segmentation.

At the start of acquisition, the framework was initialized. This included setting up the Gadgetron pipeline, importing the necessary modules, and computing trajectories, density compensation weights,^20^ coil sensitivity maps^21^ (from first 10 frames of data), and Maxwell correction terms.^22^

Figure 2 depicts the pipeline at inference. Flow‐encoded and flow‐compensated frames were gridded, coil‐combined, cropped, normalized, and buffered into 2D + time blocks. Deep artifact suppression was then performed in a sliding window fashion (window = 24 frames, step size = 18 frames, keeping only central frames to remove edge effects) separately on the flow‐encoded and compensated blocks.

The artifact‐suppressed data were then combined (average magnitude and phase subtraction), and the magnitude images were equalized using CLAHE. The magnitude and phase subtracted data were then passed to the segmentation network (as real and imaginary channels) for aortic segmentation. These segmented data were used to quantify flow from the phase‐subtracted PCMR data (after Maxwell correction). Peak detection was then performed for real‐time monitoring of heartrate, stroke volume, and CO. The resultant images, segmentation, and flow curves were displayed with low latency on the external computer (interface shown in Figure 1B).

Reconstruction timings were recorded during acquisition on the last 1000 frames. Latency is approximated as the time between the start of an acquisition block of 24 images, until completion of the reconstruction and segmentation of the block of images.

Real‐time images at rest (4–14 s) and at peak exercise (107–117 s) were additionally reconstructed offline to compare the proposed method with a CS reconstruction of the same data using temporal total variation regularization (BART^23^ toolbox). The CS regularization factor was set empirically (λ = 5E‐4), and data were reconstructed in the same blocks as the DL reconstruction (window = 24 frames, step size = 18 frames, keeping only central frames). Cardiac output was extracted for both proposed DL and CS methods using the same segmentation (obtained from the proposed DL segmentation network of the DL reconstructed images) to limit the source of discrepancies to reconstruction differences only.

Additionally, real‐time CO obtained at rest was compared with the reference standard PCMR, which was segmented using a semi‐automatic method based on an optical flow registration with manual operator correction (by an expert [J.B.]).

### 2.7 Statistical analysis

Statistical analyses were performed using Python. All compared distributions were tested for normality using Shapiro–Wilk tests. In silico metrics were compared using paired *t* tests. In vivo, Bland–Altman analysis of the prospective extracted flow volumes was performed among the reference PCMR (at rest only), FReSCO, and CS measurements. Cardiac output mean biases and limits of agreement were reported. These biases were tested for statistical significance using a repeated‐measures one‐way ANOVA (if > 2 groups) or paired *t* tests otherwise.

## 3 RESULTS

### 3.1 Training and in silico validation

Training with hyperparameter optimization of the deep artifact suppression and segmentation networks took 67 and 10 h, respectively. The range of parameters explored and final selected network parameter values for both tasks are found in Supporting Information Table S2.

In silico results are shown for 4 test subjects (including the worst test case with Dice score of 0.56) in Supporting Information Figure S3 (and 1 representative subject in Supporting Information Video S1). For deep artifact suppression, the mean absolute error, peak SNR, and average SSIM were 0.023 ± 0.004, 29.3 ± 1.4, and 0.88 ± 0.03, respectively. For the segmentation model, the binary cross entropy and Dice were 0.048 ± 0.054 and 0.87 ± 0.13. It should be noted that segmentation accuracy on restored images was not statistically significantly different from the segmentation accuracy obtained from “truth” images (binary cross entropy = 0.061, *p* = 0.09; Dice 0.87, *p* = 0.71).

### 3.2 Feasibility of proposed method

**FIGURE 3:**
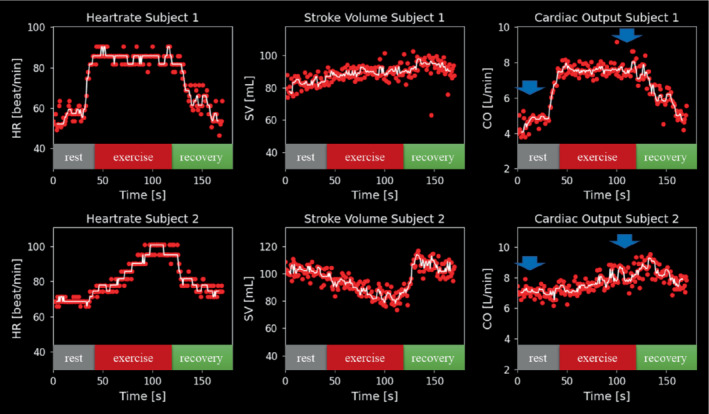
Heartrate (HR), stroke volume (SV), and cardiac output (CO) curves obtained from two subjects with different responses to exercise. In white, the median filtered curves. Blue arrows depict the 10 s areas used for comparison to CS and reference at rest (4–14 s) and CS only at peak exercise (107–117 s)

A video of the proposed interface for real‐time monitoring as recorded during scanning is provided in Supporting Information Video S2 and a snapshot of the interface at the end of acquisition in Figure 1B. The FReSCO framework was able to adequately remove artifact and segment the aorta with a latency < 1 s both at rest and during exercise without any user interaction. The CO increased from 5.82 ± 1.10 L/min at rest to 7.42 ± 1.34 L/min at peak exercise, and heartrate increased from 68 ± 8 bpm at rest to 94 ± 8 bpm at peak exercise. Two representative curves recorded during exercise are shown in Figure 3 and demonstrate different responses to exercise better seen with continuous monitoring.

A schematic of reconstruction timings is shown in Figure 2B. The gridding time was approximately 16.2 ms/frame, compared with an acquisition time of about 35 ms/frame. After the final frame in a block was gridded, deep artifact suppression of both encodings and segmentation of a block took on average of 151 ms. It should be noted that initialization of the pipeline led to an initial latency of about 16 s. However, as the total reconstruction time for a block was shorter than the acquisition time (for 18 central frames, gridding and deep artifact suppression added up to ∼443 ms vs ∼630 ms of acquisition), the reconstruction was able to catch up after 26 s (42 s into the acquisition). After this transition period, mean latency was about 622 ms for the central frame of the block, and 902 ms for the first frame.

### 3.3 Comparison with CS reconstruction and reference standard PCMR

**FIGURE 4:**
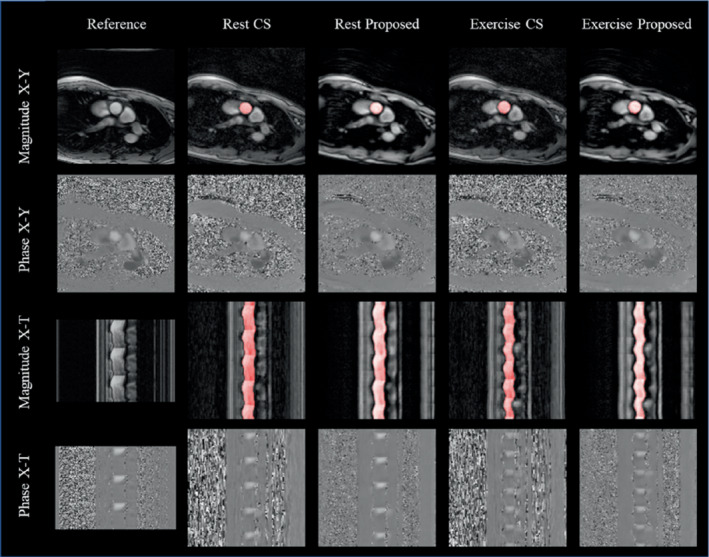
Reference (repeated for three cycles), compressed sensing (CS), and FReSCO images at rest, and CS and FReSCO images during exercise in a representative subject. Magnitude x‐y, magnitude x‐t, phase x‐y, and phase x‐t images are shown. Automatic segmentations (computed from the proposed deep learning [DL] images) are overlaid on top of the magnitude images. Corresponding video in Supporting Information Video S3

**FIGURE 5:**
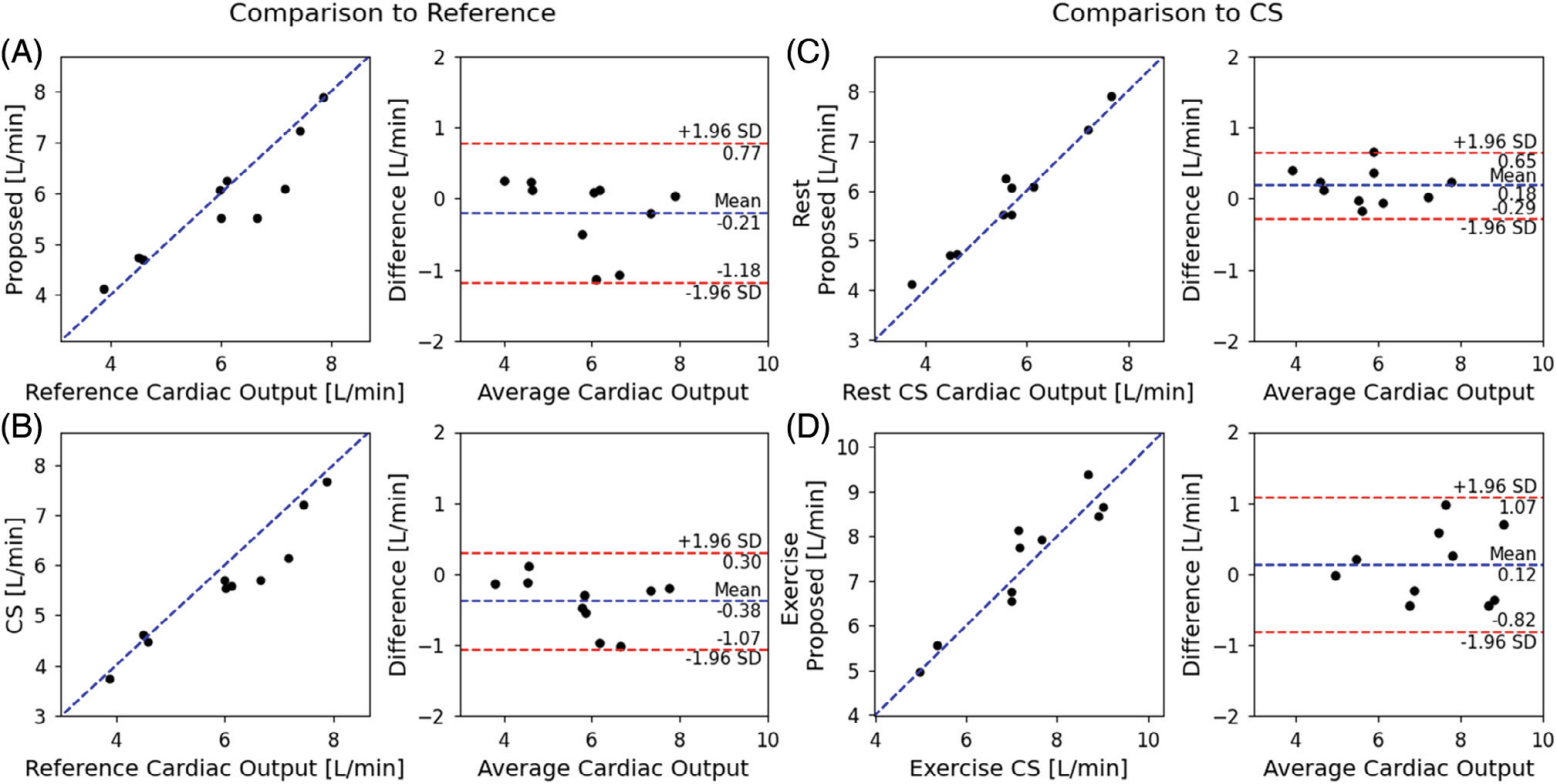
Correlation and Bland Altman plots comparing cardiac output from proposed FReSCO to reference at rest (A), compressed sensing (CS) to reference at rest (B), FReSCO to CS at rest (C), and FReSCO to CS at peak exercise (D). The values reported for CS and FReSCO are the average cardiac output obtained over 4 to 14 s (rest) and 107 to 117 s (exercise) of the real‐time acquisition

Magnitude and phase‐subtracted images, reconstructed using FReSCO and CS, as well as reference ECG‐gated data, are shown in Figure 4 (Supporting Information Video S3). Time‐averaged and real‐time curves are shown for 1 subject in Supporting Information Figure S4 (blood flow) and Supporting Information Figure S5 (mean velocity and segmentation area). At rest, there was good agreement between the proposed method and the reference (Figure 5A), with no significant differences in CO (bias = −0.21 ± 0.50 L/min, *p* = 0.246). There was a small but statistically significant negative bias in CO between CS and the reference (bias = −0.38 ± 0.35 L/min, *p* = 0.009; Figure 5B) and a trend toward significance between the proposed DL method and CS (bias = 0.18 ± 0.24 L/min, *p* = 0.052; Figure 5C). During exercise, there was good agreement in peak CO between CS and FReSCO (bias = 0.12 ± 0.48 L/min, *p* = 0.458; Figure 5D).

## 4 DISCUSSION

In this study, we demonstrated the feasibility of low‐latency real‐time CO monitoring using real‐time PCMR, combined with DL‐based artifact suppression and automated segmentation during exercise. There was good agreement between CO measured using this method and conventional gated PCMR (at rest), and CS reconstruction of the same data (at rest and exercise). Thus, we believe our approach has the potential to simplify continuous CO measurement during various stress protocols.

### 4.1 Networks and training

We have previously shown the utility of deep artifact suppression for fast reconstruction of highly undersampled spiral real‐time PCMR.^10^ As our application also required processing of PCMR data, we additionally trained a network for fully automated segmentation. We showed excellent network performance for both tasks, and the combined inference time was short enough for a latency < 1 s. Thus, more sophisticated joint reconstruction and segmentation networks^24^, ^25^ that could have had shorter inference times were not necessary. Further benefits of splitting the tasks were that it allowed the use of a larger data set for learning deep artifact suppression, different preprocessing of the inputs (eg, CLAHE before segmentation), and separate optimization of network hyperparameters.

Our labeled data set was relatively large, using 70 data sets for training the segmentation. Preliminary results showed that similar performance could be obtained with as few as 10 training data sets (violin plots and qualitative comparisons are shown in Supporting Information Figures S6 and S7), which might indicate that less time‐consuming labeling is required. However, failure in segmentation was observed in the in silico data, potentially due to unusual anatomy, image artifacts, or position of the aorta in the FOV.

Future improvements could include using multicoil raw data for training^26^ and using a more end‐to‐end approach to optimize acquisition, reconstruction,^27^, ^28^, ^29^ and segmentation.^24^ However, iterative model‐based approaches would require significant changes/optimization to provide reasonable latencies, as each data‐consistency step requires two additional nonuniform fast Fourier transforms (taking 16.2 ms each in our current framework); thus, one iteration would take 48.6 ms, which is already much larger than the acquisition time (∼35 ms).

### 4.2 Real‐time reconstruction and visualization

The proof‐of‐concept framework was demonstrated at the scanner during continuous exercise. It was able to remove artifacts and segment images without user intervention and with low enough latency to provide almost real‐time monitoring. Latency could be further reduced by including (1) initialization of the pipeline before the start of acquisition to reduce the initial latency, (2) parallelization of gridding and deep artifact suppression for higher frame rate, and (3) using a memory‐based network to reconstruct the latest frame rather than blocks, while still using temporal redundancies.^30^

### 4.3 Comparison with CS reconstruction and gated reference standard

There was reasonable agreement between FReSCO and the reference standard ECG‐gated PCMR sequence for measurement of CO at rest. This was despite FReSCO's lower resolutions required for real‐time imaging and higher velocity encoding necessary for exercise studies. There was also reasonable agreement between the proposed DL and CS reconstructions of the same data at rest and exercise. This is in keeping with previous larger studies comparing deep artifact suppression to CS and gated PCMR. However, in our case, agreement with reference PCMR also relied on the accuracy of automated segmentation. The good agreement suggests that both deep artifact suppression and automated segmentation worked robustly within our framework.

Beat‐to‐beat curves showed variations of CO (Figures 1B and 3). We believe that this variability could be due to physiological variations in stroke volume due to respiration, which could be a relevant biomarker in itself.^31^ It could also be due to remaining inaccuracies in segmentation and reconstruction. However, Supporting Information Figure S5 shows plausible physiological changes in beat‐to‐beat mean velocity and area without obvious segmentation or reconstruction failures.

Nevertheless, further testing in larger and more heterogenous populations is required before more general use.

### 4.4 Applications

Stress testing, particularly with exercise, is becoming increasingly important in cardiac MRI, as it provides important information about hemodynamic responsiveness. However, it has been difficult to continuously monitor CO due to long reconstruction times and difficulty in segmenting thousands of frames of PCMR data. We have demonstrated this is easily achieved with our framework, and we showed that the dynamic response to exercise was highly variable.

Thus, continuous CO data may provide new insights into cardiovascular disease. In future work we aim to assess the feasibility of this approach in more strenuous forms of in‐scanner exercise (eg, recumbent bicycle).

## 5 CONCLUSIONS

The FReSCO framework enables real‐time monitoring of CO during exercise and could greatly simplify workflow and provide a convenient tool for assessment of the hemodynamic response to a range of stressors (eg, exercise, adenosine, dobutamine, eating, mental tasks). Future work will aim to generalize the framework to multiple vessels of interest and use the proposed framework within research protocols.

## Supporting information

 
**Figure S1.** Left: Example of variable‐density golden angle spiral trajectory for one cardiac phase image. Three spiral arms rotated by the golden angle are combined for each cardiac phase with a golden angle increment. The same spiral arm is acquired twice consecutively with and without flow encoding. Each spiral arm is accelerated by 26 × in the center 20% of k‐space and 65 × in the outermost 20% of k‐space. Right: Trajectories covered in two consecutive cardiac phases. Each frame is accelerated by 8.7 × in the center 20% of k‐space and 21.7 × in the outermost 20% of k‐space
**Figure S2.** Diagram showing the U‐Net architecture and explored hyperparameters (in red). Range explored and final selected parameter values for both tasks are provided in Table S1
**Table S1.** Information relative to the retrospective (deep artifact suppression and segmentation) and prospective cohorts and studies
**Table S2.** A, Information relative to the data augmentation and training of individual networks. B, Resulting values selected from the hyperband optimization as well as the range explored for each hyperparameter
**Figure S3.** Top to bottom: Four test subjects (including worst Dice score on the restoration + segmentation task). Left to right: Ground‐truth images and segmentation, ground‐truth images and DL segmentation calculated from ground‐truth images, undersampled images (input to deep artifact suppression network) and DL restored images, and DL segmentation (estimated from DL images). The segmentations are overlaid in red when applicable
**Figure S4.** Left: Averaged FReSCO, averaged compressed sensing (CS), and reference flow curves. Right: Real‐time FReSCO and CS flow curves obtained at rest (A) and during exercise (B) showing good agreement between methods
**Figure S5.** Left: Averaged FReSCO, averaged CS, and reference mean velocity and area curves. Right: Real‐time FReSCO and CS mean velocity and area curves obtained at rest (A) and during exercise (B)
**Figure S6.** Violin plots showing test‐set segmentation Dice scores obtained from restored images for models trained using 70 (used model), 50, 20, 10, and 5 training data sets. The segmentation quality was significantly lower only when using 5 training data sets when compared with using 70 data sets. *Statistically significant; n.s., not statistically significant
**Figure S7.** Representative test case. From left to right: Top: Ground‐truth images and overlaid segmentations from the original data set and from the models trained with 70, 50, 20, 10, and 5 data sets. Bottom: Input images, denoised images, and overlaid segmentations from best models trained with 70, 50, 20, 10, and 5 data sets

**Video S1.** Representative test‐set subject. Top row: Magnitude ground‐truth images and segmentation, overlaid predicted segmentation from ground truth, undersampled input, restored images, and overlaid predicted segmentation from restored images. Bottom row: Matching phase images

**Video S2.** Real‐Time flow monitoring during exercise. Interface during the start of exercise, peak exercise, and end of recovery (40–50, 110–120, and 170–180 s). Top row: Magnitude and overlaid segmentation, phase, and extracted blood flow curve with marked detected peaks. Bottom row: Beat‐to‐beat heartrate, stroke volume, and cardiac output as provided in real time

**Video S3.** Comparison depicting the magnitude, segmentations, and flow maps for reference, CS at rest, FReSCO at rest, CS at exercise, and FReSCO at exercise of the same subject

## Data Availability

Code is available from the corresponding author upon reasonable request.
