# Supplementary material for: FReSCO: Flow Reconstruction and Segmentation for low‐latency Cardiac Output monitoring using deep artifact suppression and segmentation
Source: Magn Reson Med. 2022 Jul 4;88(5):2179–89. doi: 10.1002/mrm.29374 (PMC9545927; doi:10.1002/mrm.29374)
Supplement: Supplementary file 1 — Figure S1. Left: Example of variable‐density golden angle spiral trajectory for one cardiac phase image. Three spiral arms rotated by the golden angle are combined for each cardiac phase with a golden angle increment. The same spiral arm is acquired twice consecutively with and without flow encoding. Each spiral arm is accelerated by 26 × in the center 20% of k‐space and 65 × in the outermost 20% of k‐space. Right: Trajectories covered in two consecutive cardiac phases. Each frame is accelerated by 8.7 × in the center 20% of k‐space and 21.7 × in the outermost 20% of k‐space Figure S2. Diagram showing the U‐Net architecture and explored hyperparameters (in red). Range explored and final selected parameter values for both tasks are provided in Table S1 Table S1. Information relative to the retrospective (deep artifact suppression and segmentation) and prospective cohorts and studies Table S2. A, Information relative to the data augmentation and training of individual networks. B, Resulting values selected from the hyperband optimization as well as the range explored for each hyperparameter Figure S3. Top to bottom: Four test subjects (including worst Dice score on the restoration + segmentation task). Left to right: Ground‐truth images and segmentation, ground‐truth images and DL segmentation calculated from ground‐truth images, undersampled images (input to deep artifact suppression network) and DL restored images, and DL segmentation (estimated from DL images). The segmentations are overlaid in red when applicable Figure S4. Left: Averaged FReSCO, averaged compressed sensing (CS), and reference flow curves. Right: Real‐time FReSCO and CS flow curves obtained at rest (A) and during exercise (B) showing good agreement between methods Figure S5. Left: Averaged FReSCO, averaged CS, and reference mean velocity and area curves. Right: Real‐time FReSCO and CS mean velocity and area curves obtained at rest (A) and during exercise (B) Figure S6. Violin plots showing test‐se [file MRM-88-2179-s003.docx]

Supporting Information

FReSCO: Flow Reconstruction and Segmentation for low latency Cardiac Output monitoring using deep artifact suppression and segmentation


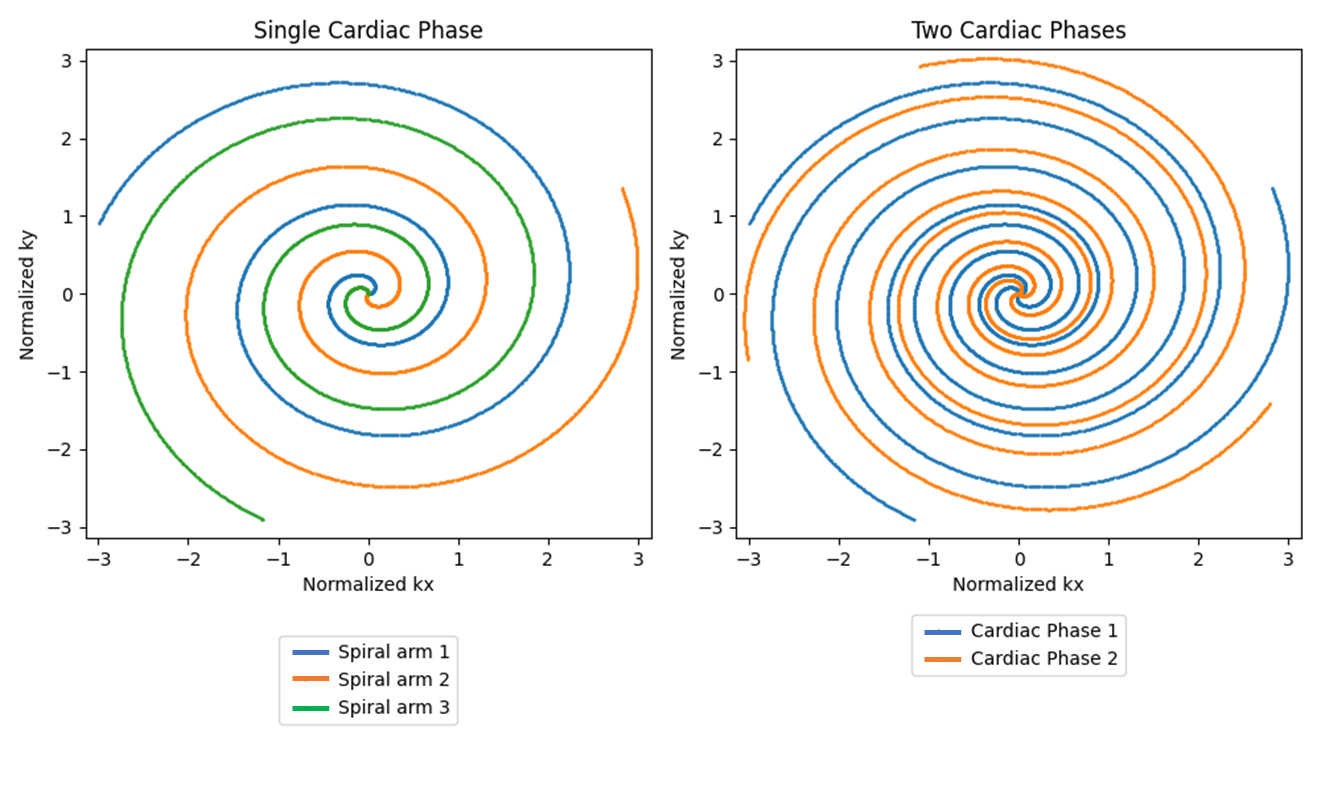


**Supporting Information Figure S1.** Left: Example of variable density golden angle spiral trajectory for one cardiac phase image. Three spiral arms rotated by the golden angle are combined for each cardiac phase with a golden angle increment. The same spiral arm is acquired twice consecutively with and without flow encoding. Each spiral arm is accelerated by x26 in the center 20% of k-space and x65 in the outermost 20% of k-space. Right: Trajectories covered in two consecutive cardiac phases. Each frame is accelerated by x8.7 in the center 20% of k-space and x21.7 in the outermost 20% of k-space.


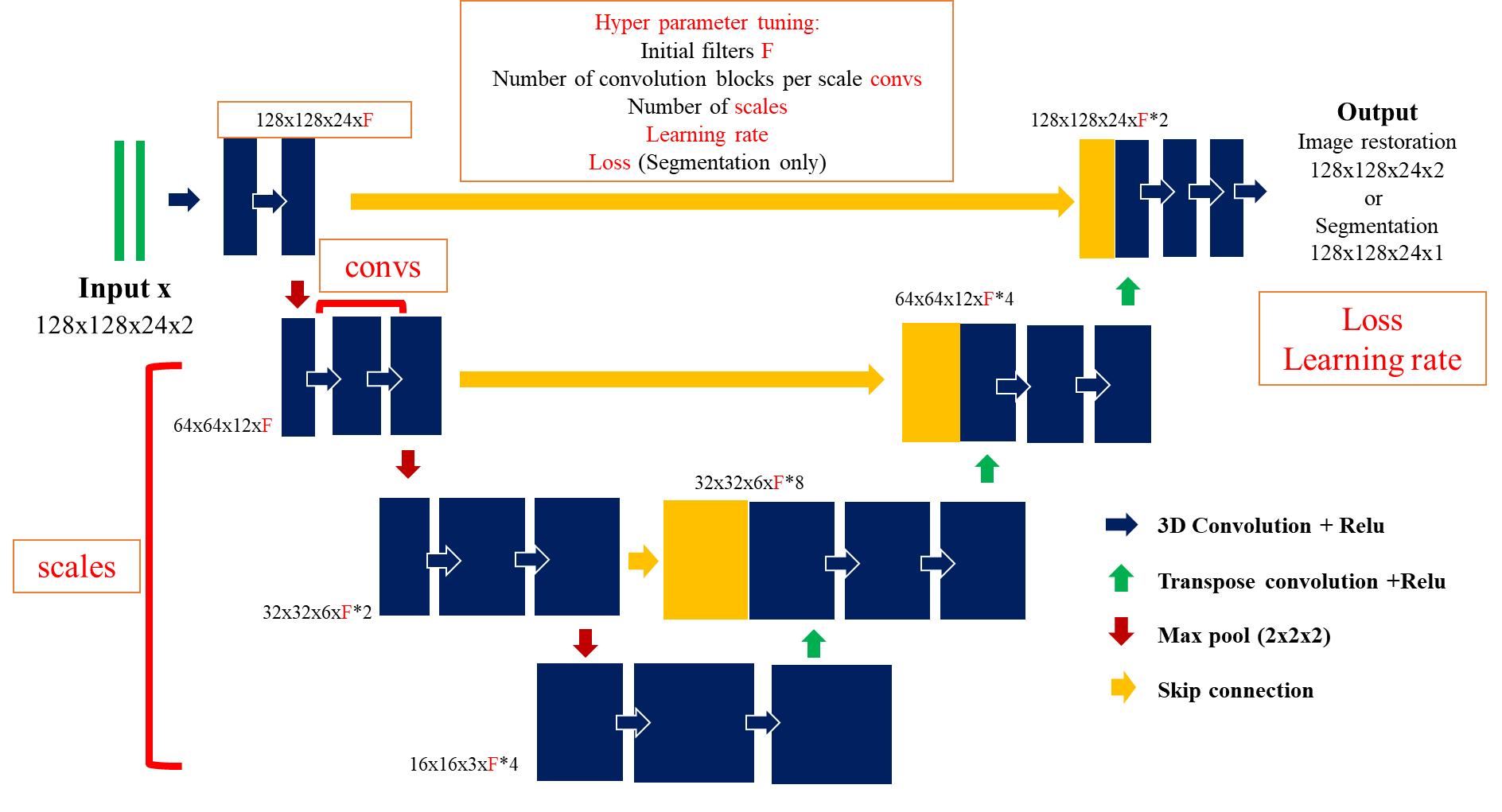


**Supporting Information Figure S2.** Diagram showing the U-Net architecture and explored hyper parameters (in red). Range explored and final selected parameter values for both tasks are shown in Supporting Information Table S1.

*
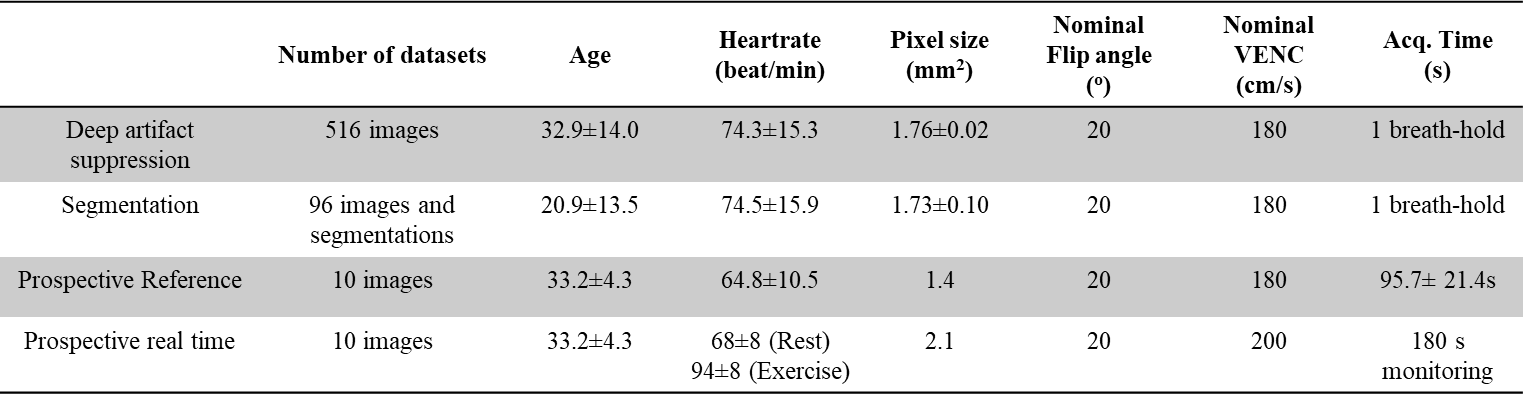
*

**Supporting Information Table S1.** Information relative to the retrospective (Deep artifact suppression and Segmentation) and prospective cohorts and studies.


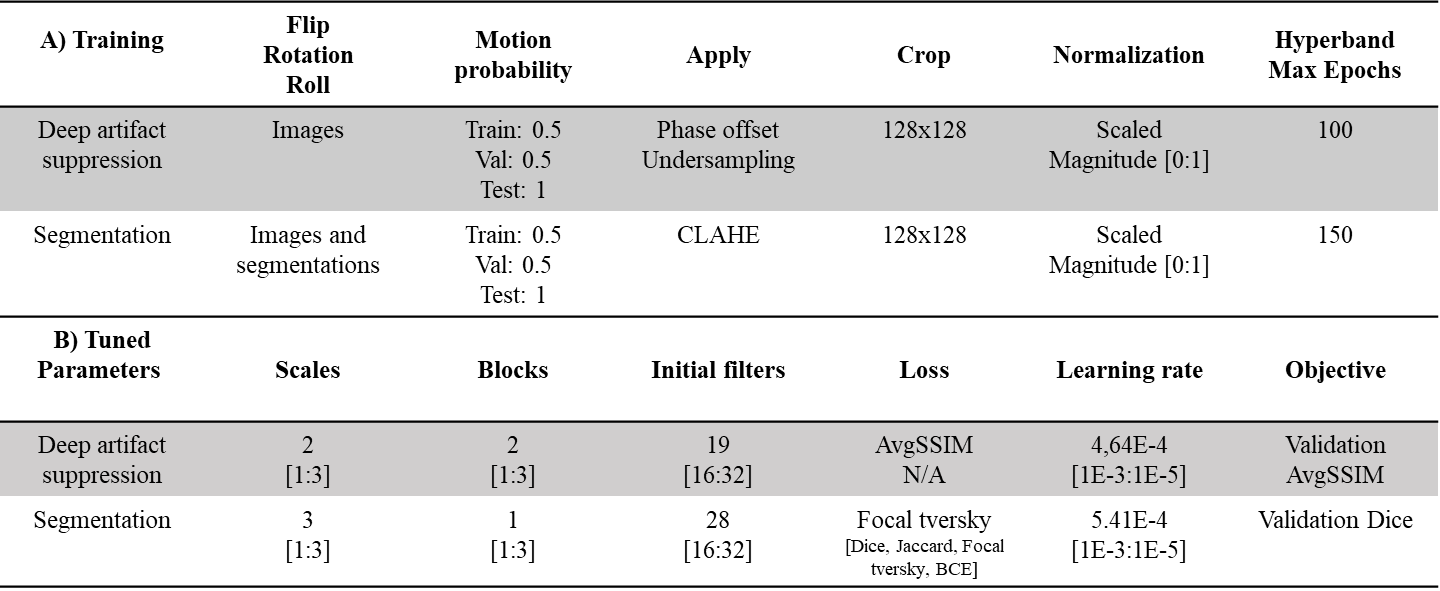


**Supporting Information Table S2.** A) Information relative to the data augmentation and training of individual networks. B) Resulting values selected from the hyperband optimization as well as the range explored for each hyper-parameter.


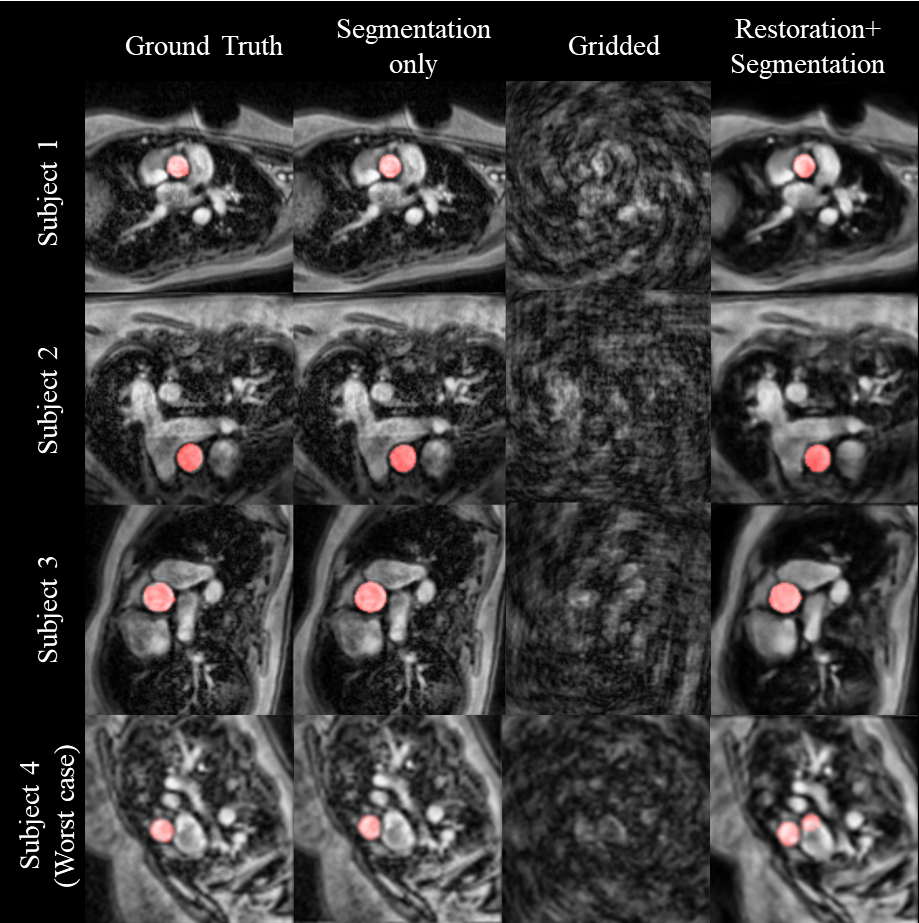


**Supporting information Figure S3.** Top to Bottom: Four test subjects (including worst Dice score on the restoration + segmentation task). Left to right: Ground Truth images and segmentation, Ground truth images and DL segmentation calculated from Ground Truth images, undersampled images (input to deep artifact suppression network) and DL restored images and DL segmentation (estimated from DL images). The segmentations are overlaid in red when applicable.


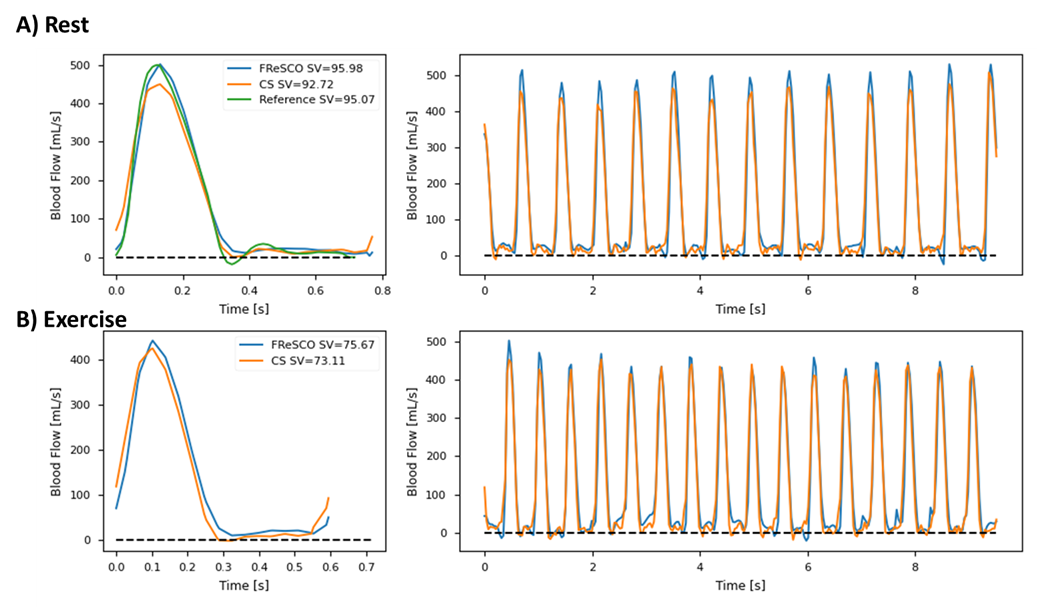
**Supporting Information Figure S4.** Left: Averaged FReSCO, averaged CS and reference flow curves and Right: real-time FReSCO and CS flow curves obtained A) at rest and B) during exercise showing good agreement between methods.


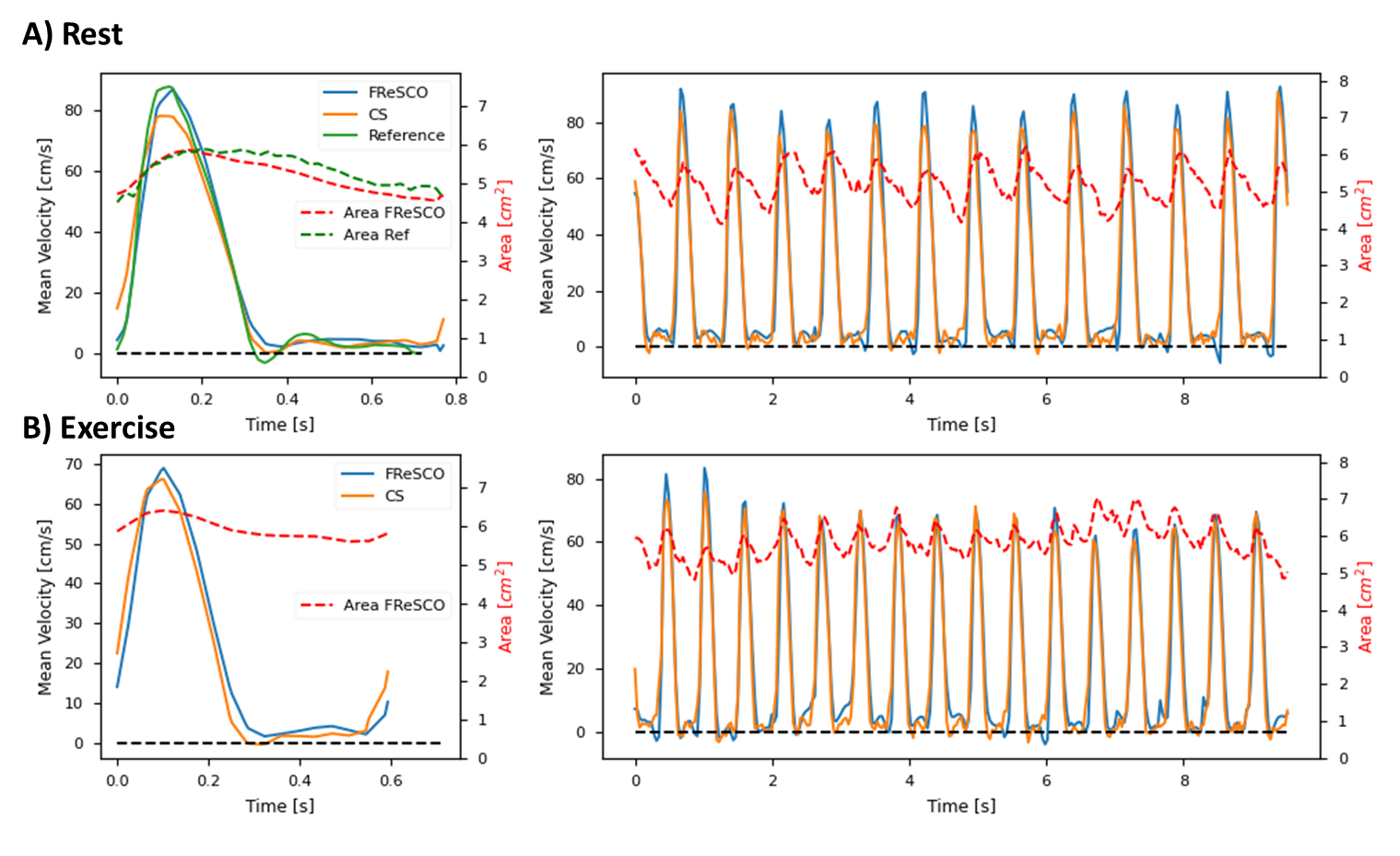
**Supporting information Figure S5.** Left: Averaged FReSCO, averaged CS and reference mean velocity and area curves and Right: real-time FReSCO and CS mean velocity and area curves obtained A) at rest and B) during exercise.


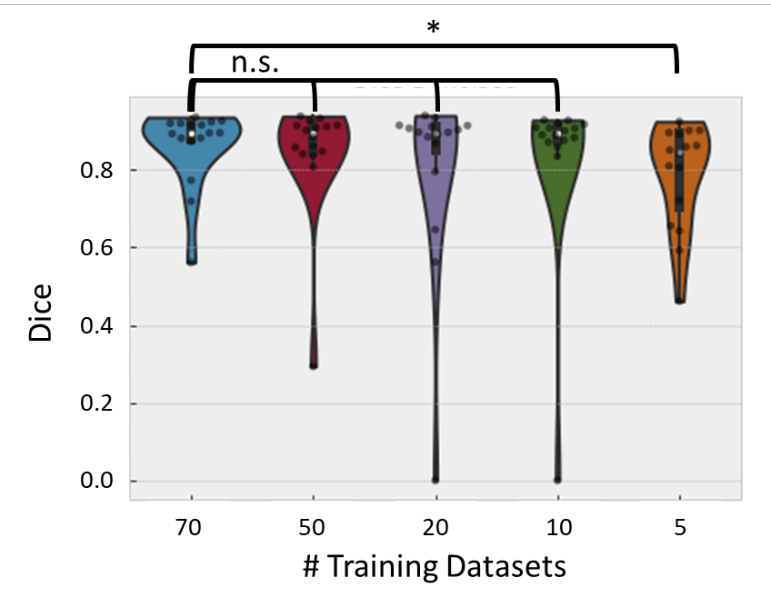


**Supporting Information Figure S6.** Violin plots showing test set segmentation Dice scores obtained from restored images for models trained using 70 (used model), 50, 20, 10 and 5 training datasets. The segmentation quality was significantly lower only when using 5 training datasets when compared to using 70 datasets. *: statistically signicant, n.s.: not statistically significant.


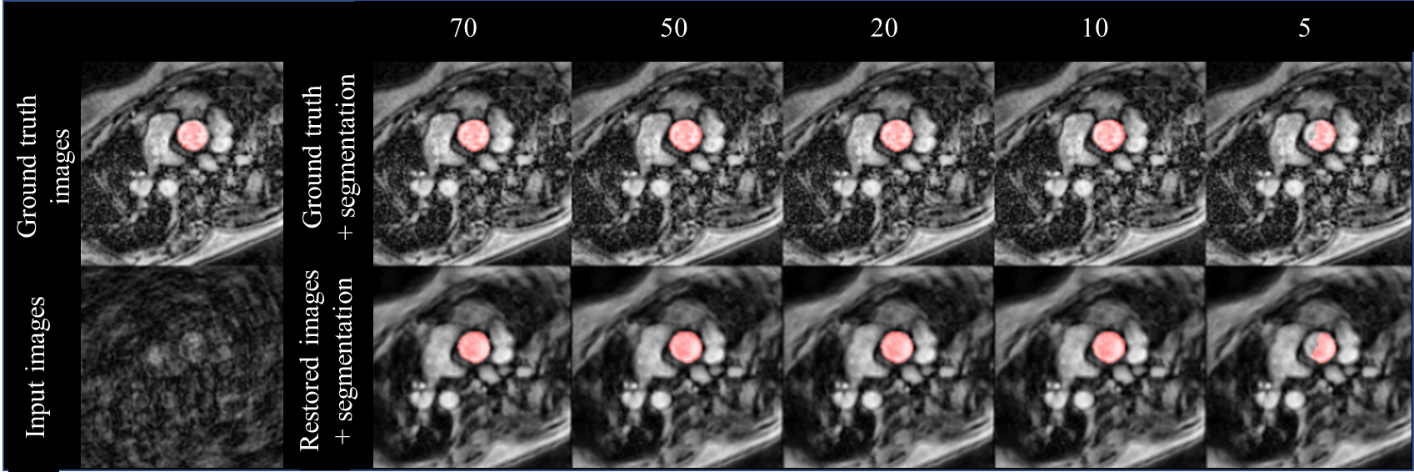


**Supporting Information Figure S7.** Representative test case. From left to right: Top: Ground truth images and overlaid segmentations from the original dataset and from the models trained with 70, 50, 20, 10 and 5 datasets. Bottom: Input images and Denoised images and overlaid segmentations from best models trained with 70, 50, 20, 10 and 5 datasets.

**Supporting Information Video S1.** Representative test set subject Video. Top row: Magnitude ground truth images and segmentation, overlaid predicted segmentation from ground truth, undersampled input, restored images and overlaid predicted segmentation from restored images. Bottom row: Matching phase images.

**Supporting Information Video S2. Real-Time flow monitoring during exercise.** Video of the interface during the start of exercise, peak exercise and end of recovery (seconds 40-50, 110-120 and 170-180) Top Row: Magnitude and overlaid segmentation, Phase and extracted blood flow curve with marked detected peaks. Bottom Row: Beat-to-beat heartrate, stroke volume and cardiac output as provided in real-time.

**Supporting Information Video S3.** Comparison video depicting magnitude, segmentations and flow maps for Reference, CS at rest, FReSCO at rest, CS at exercise and FReSCO at exercise of the same subject.
